# Does COVID-19 impact the QT interval prolongation? Answers from genetic causal inference

**DOI:** 10.1042/BSR20241281

**Published:** 2025-01-22

**Authors:** Yongfei Song, Zequn Zheng

**Affiliations:** 1 Ningbo Institute of Innovation for Combined Medicine and Engineering, Ningbo Medical Center Lihuili Hospital, Ningbo University, Ningbo, Zhejiang, China; 2 Department of Cardiology, First Affiliated Hospital of Shantou University Medical College, Shantou, Guangdong, China

**Keywords:** Causality, COVID-19, Long QT Syndrome, Mendelian Randomization Analysis

## Abstract

During the COVID-19 pandemic, there has been heightened interest in the QT interval, a crucial indicator of ventricular electrical activity. Mendelian randomization (MR) is used here to investigate the genetic causation between QT interval alterations and COVID-19. Genetic proxies representing three COVID-19 phenotypes—severe, hospitalized, and COVID-19—were identified in over 1,000,000 individuals of European ancestry. Univariate two-sample MR (TSMR) and multi-exposure-adjusted multivariate MR (MVMR) were used to assess genetic causal associations between COVID-19 and QT intervals in 84,630 UK Biobank participants. The MR-robust adjusted profile score (MR-RAPS) method and radial MR frame were utilized for effective robustness and outlier variant detection, with sensitivity analyses conducted to identify horizontal pleiotropy. For every COVID-19 phenotype, univariate TSMR analysis revealed non-significant causal estimates between COVID-19 and the QT interval [COVID-19: β_IVW_ (95% CI): −0.44 (−1.72, 0.84), *P* = 0.50; hospitalization: β_IVW_: 0.12 (−0.57, 0.80), *P* = 0.74; severe case: β_IVW_: 0.11 (−0.29, 0.51), *P* = 0.58]. MR-RAPS and outlier-corrected radial MR analyses further supported this null causal estimation. In confounder-adjusted MVMR analysis, this nonsignificant causality was independent of body mass index (BMI), smoking, and alcohol consumption [β_BMI+Alcohol+Smoking_ (95% CI): −0.77 (−2.44, 0.91), *P* = 0.37]. Sensitivity analyses did not detect any evidence of bias from horizontal pleiotropy, abnormal data distribution, or weak instruments. These findings suggest that COVID-19 does not directly causally prolong the QT interval. Inconsistent findings in observational research may be attributed to residual confounding.

## Introduction

Cardiovascular diseases continue to be a significant global cause of mortality, and the severe acute respiratory syndrome coronavirus 2 (SARS-CoV-2)-induced COVID-19 has exacerbated this burden [1–3]. COVID-19 exerts a multi-organ, multi-level impact on the human body, with clinical ailments varying from instances with no symptoms to severe respiratory distress with a fatal outcome [4]. Notably, the virus extends its influence beyond the respiratory system, resulting in a spectrum of cardiovascular complications, including a pronounced association with various cardiac arrhythmias, which has led to an increase in hospitalization rates and mortality [2,3,5–7].

Among the array of cardiovascular concerns arising from COVID-19, the modulation of QT interval on electrocardiogram (ECG) has garnered substantial attention [8–16]. QT interval, a vital parameter measured on ECGs, reflects the time required for ventricular depolarization and repolarization, which is influenced by a multitude of factors, including heart rate, age, gender, medications, electrolytes, and hormones [17]. Prolonged or shortened QT intervals increase sudden cardiac death risk by potentially triggering severe arrhythmias such as ventricular tachycardia, especially torsades de pointes [18,19].

ECG serves as an essential tool in managing COVID-19 patients, unveiling heart involvement such as atrial fibrillation, prolonged QT intervals, and ventricular and supraventricular arrhythmias [20]. Numerous observational studies and case reports have indicated a potential link between COVID-19 and longer QT intervals on ECG [5,9,14–16,21].

Despite increasing research identifying genetic loci linked to COVID-19 susceptibility and severity, the connection between these genetic markers and adverse outcomes in patients remains unclear [7,26–28]. From a biological standpoint, although QT interval prolongation may be associated with SARS-CoV-2-induced myocarditis and changes in physiological parameters such as electrolytes and hormones [8,29], the observational and cross-sectional nature of these studies, coupled with the complexity of this relationship influenced by multiple factors, leave us uncertain as to whether a genuine causal association exists between SARS-CoV-2 infection and QT interval prolongation.

To determine if COVID-19 directly influences QT interval changes, we employed Mendelian randomization (MR) analysis, a statistical strategy that uses genetic variations as instruments to identify causal links between risk factors and health outcomes, reducing biases typical in observational studies [30]. We adopted genetic variants related to COVID-19 exposure as instrumental, employing both univariable MR (UVMR) and multivariable MR (MVMR) analyses to assess their impact on QT intervals, emphasizing the importance of establishing causation rather than mere correlation.

## Materials and methods

### Study design

We categorized the COVID-19 phenotypes into three groups: general infection, hospitalization, and severe cases requiring respiratory support, each compared against the general population. Based on the MR study design, we initially selected COVID-19-associated significant genetic variants (single nucleotide polymorphisms, SNPs) from each COVID-19 phenotype’s genome-wide association study (GWAS) to serve as instrumental variables (IVs) for causal inference. These IVs met the three core MR assumptions: strong association, genetic independence, and exclusion restriction. Eligible SNPs were harmonized with QT interval GWAS data to ensure alignment of effect directions. Subsequently, we estimated the genetic risk of SNP-related exposure on QT interval changes using univariable two-sample MR (TSMR) through the TwoSampleMR software package. Additionally, we adjusted for potential confounders, including body mass index (BMI), smoking, and alcohol consumption, by applying MVMR using the MVMR package. To verify the robustness of our causal findings, we conducted a series of sensitivity analyses, including heterogeneity testing, pleiotropy analysis, leave-one-out tests, normality checks, and identification of influential outliers that might affect causal relationships.

### Genetic summary statistics of COVID-19, BMI, alcohol, and smoking

Summary data of three COVID-19 phenotypes were derived from the COVID-19 Host Genetics Initiative repository (https://www.covid19hg.org) (round 7, released on April 8, 2022), which integrates dozens of cohort samples including general infection, hospitalization, and severe cases necessitating respiratory support [31]. Ethical approval and demographic adjustments were noted for the data, which includes 122,616 cases of COVID-19 infection, 32,519 hospitalized cases, and 13,769 severe cases, compared with 2,475,240, 2,062,805, and 1,072,442 controls, respectively. Additional GWAS summary statistics for BMI, alcohol consumption, and smoking were available at the IEU Open GWAS Project (http://gwas.mrcieu.ac.uk). These datasets comprise 681,275 samples for BMI (ID: ieu-b-40), 462,346 for alcohol consumption (ID: ukb-b-5779), and 468,170 for smoking (ID: ebi-a-GCST90029014).

### GWAS summary statistics for QT interval

The summary statistics for the QT interval on ECG were derived from recent large-scale GWASs from Nauffal et al. (84,630 UK Biobank participants) [32,33]. The QT interval was extracted from 12-lead or 3-lead ECGs and adjusted for heart rate using the Bazett formula: QTc = $QT(ms)/\sqrt{RR}(s)$. Data of low quality and potential confounding factors influencing QT intervals were excluded from the study, and further details regarding data quality control can be found in the original publication [32]. GWAS summary data for the study are accessible on the Cardiovascular Disease Knowledge Portal (CVDKP) [32]. SNPs used as genetic instruments for COVID-19 exposure were matched with these QT interval data for primary MR analysis [34]. For replication, we used QT interval GWAS data from Hoffmann et al., which included 86,165 participants (73.3% European ancestry; study accession GCST90165291, available on the NHGRI-EBI GWAS Catalog) [33]. These QT interval outcome data (QT*_Hof_*) were employed for replication analysis to verify the consistency of the primary MR outcomes.

### Identification of genetic IVs

In each COVID-19 GWAS, SNP-assigned genome-wide significance (*P*<5 default 10^−8^) were designated as genetic IVs [34]. These significant subsets were extracted from each COVID-19 GWAS. To ensure suitability for causal inference under the MR framework, we required that these IVs be strongly associated with COVID-19, unrelated to QT interval directly, and independent of potential confounders. Subsequently, we applied linkage disequilibrium (LD) clumping (r² < 0.001, 10,000 kb window) on SNP data to confirm the genetic independence of the selected IVs [34]. Additionally, we used PhenoScanner to exclude IVs directly related to possible confounders (especially associated with QT intervals) such as BMI, diabetes, tobacco and alcohol consumption, hypertension, cholesterol, medication influencing QT interval, mental disorders, and insomnia [35]. To assess the association strength of each included IV and reduce potential weak instrument bias, we calculated the F statistic for each IV as [(effect size(β)/standard error of effect size (β_SE_) [2,34]. For visual representation, regional association plots of the lead SNPs (IVs with the smallest *P-*values) were generated using LocusZoom (http://csg.sph.umich.edu/locuszoom/), concerning the combined hg19/1000 Genomes EUR panel.

### Statistics analysis

To evaluate bias and Type I error rates, we used the tool provided by Burgess et al. (https://sb452.shinyapps.io/overlap/), which estimates these metrics under the null hypothesis [36]. By inputting the sample sizes for both the exposure and outcome, we obtained the necessary estimates to evaluate the overlap proportion using default parameters. For causal effect estimation with overlapping samples, we used the MRlap package (version 0.0.3.2). MRlap function adjusts for sample overlap and corrects biases like weak instrument bias and the winner’s curse using summary-level data, ensuring more accurate and reliable results [37]. The Medical Research Council Integrative Epidemiology Unit (MRCIEU) TwoSampleMR software package (version 0.6.2) was used to harmonize the effect directions across exposure and outcome SNPs using the harmonise_data function with the setting action = 2. The package’s mr function was then employed to perform MR causal analysis [38]. Pooled exposure-outcome causal effects determined by univariable MR (UVMR) in the mr function were estimated using inverse variance weighted (IVW) (multiplicative random effects) methods, coupled with MR-Egger, weighted median, and penalized weighted median [34]. MR-robust adjusted profile score (MR-RAPS) through mr.raps package (version 0.4.1) was further adopted for the estimation of causal effects and conducted a normality test [39]. In addition, a radial MR framework (package version 1.1) was applied, which avoids data re-orientation and employs radial plots to better discern correlations and causal relationships [40].

The MVMR analysis using the MVMR package (version 0.4) directly estimated the specific associations between each exposure and the respective outcomes and potentially reflected the pleiotropic effects of the included IVs, effectively mitigating the interference of confounding factors on observed results [41]. This analysis was achieved using the MR-PRESSO package (version 1.0) [42]. To facilitate clinical interpretation, the effect size representing the impact of COVID-19 on QT interval duration was quantified in milliseconds, reflecting how the disease presence influenced the QT interval length on an ECG. For UVMR estimation, a *P*-value<0.05 was considered indicative of a potential causal relationship. A Bonferroni multiple correction threshold of *P*<0.01 (0.05/4) was deemed statistically significant in MVMR [34].

### Sensitivity analyses

Sensitivity analyses involved evaluating heterogeneity among the IVs by applying Cochran’s Q-statistic in both the IVW and MR-Egger methods. The Q-statistic tests for variation among the IVs beyond what would be expected by chance, indicating potential heterogeneity [34]. A leave-one-out approach was also implemented to ensure that no single SNP disproportionately influenced the aggregate findings [43]. Egger intercept check generates *P*-values over 0.05 suggesting the absence of horizontal pleiotropy [43]. MR-RAPS, which proposes a consistent and asymptotically normal estimator by adjusting the profile score, was utilized to aid in interpreting potential pleiotropic effects and enhance the accuracy of causal effect estimations [39]. Furthermore, radial MR was used in the context of IVW or MR-Egger to better identify and adjust for outliers and influential observations, thereby facilitating a more accurate MR analysis [40].

## Results

### Genetic IVs for MR analysis

Following meticulously applying stringent criteria to ensure relevance and independence, we identified sets of SNPs from the COVID-19, hospitalized COVID-19, and severe COVID-19 GWAS cohorts, totaling 15, 33, and 29 variables, respectively. Regional association plots were created to visualize the chromosomal positions of the lead SNPs for each exposure (Supplementary Figure S1). These SNPs, as IVs for exposure, were compatible and harmonized with the outcome GWAS dataset (including QT_(*Hof*)_ as a duplicate analysis) for further MR assessment. The IVs were evaluated for strength to minimize weak instrument bias, using F-statistics as the measure. With values ranging from 27.04 to 845.47, all IVs exceeded the standard threshold of 10, confirming strong associations with the exposure. This high range of F-statistics ensures robust instrument strength, reducing the risk of weak instrument bias and enhancing the reliability of our MR analysis and causal inference.

### Bias estimation for potential sample overlap

Given the potential for overlapping samples, we initially assessed the Type I error rate and bias across varying degrees of overlap (from 0.0 to 1.0) to ensure the robustness of our statistical approach. In all scenarios, the Type I error rate was consistently maintained at 0.05, regardless of overlap proportion, indicating that the statistical tests we employed effectively controlled the Type I error rate at the expected level (*α* = 0.05). This result suggests that the presence of overlapping samples did not inflate the Type I error, supporting the reliability of our findings. Furthermore, the bias was recorded as 0.000 when assessing all overlap proportions, demonstrating that the estimation procedure was unbiased under all tested overlap conditions. We then performed MRlap analysis to examine the potential causal relationship between COVID-19 exposure and QT interval changes. For general COVID-19 exposure, the observed effect on the QT interval was *β* = −0.17 (*P*=0.31). After applying the correction for overlapping samples, the effect remained stable (*β* = −0.20, *P*=0.31), and the difference between the observed and corrected effects was non-significant (*P*=0.25). This consistency in the observed and corrected results suggests that overlapping samples did not significantly impact the estimated effect size for COVID-19 exposure on QT interval, confirming the robustness of our findings. Similarly, for COVID-19 hospitalization, the observed effect was *β* = 0.13 (*P*=0.28), which remained virtually unchanged after correction (*β* = 0.14, *P*=0.29), with a non-significant difference between them (*P*-difference = 0.48). The genetic correlation (rg) calculated through LD score regression was 0.15, further suggesting a weak genetic association between hospitalization due to COVID-19 and QT interval changes. For severe COVID-19 cases, the observed effect was *β* = 0.07 (*P*=0.40), which also remained consistent after correction (*β* = 0.08, *P*=0.41), with no significant difference (*P*-difference = 0.64) and a rg of 0.13. These results indicate that even for severe COVID-19 cases, the overlap did not introduce bias into our causal estimates (Table 1).

**Table 1:** TSMR analysis using potentially overlapping sample through MRlap.

Exposure	Outcome	Observed effect		Corrected effect		Difference	LD score
		Beta	*P-*value	Beta	*P-*value	*P-*value	Rg
COVID-19	QT interval	−0.17	0.31	−0.20	0.31	0.25	0.13
Hospitalization		0.13	0.28	0.14	0.29	0.48	0.15
Severe case		0.07	0.40	0.08	0.41	0.64	0.13
COVID-19	QT interval (*Hof*)	0.40	0.02	0.47	0.02	0.05	−0.04
Hospitalization		0.03	0.84	0.03	0.82	0.73	−0.04
Severe case		0.06	0.32	0.08	0.30	0.21	−0.03

### Causal estimation of genetically determined COVID-19 on QT interval

**Figure 1:**
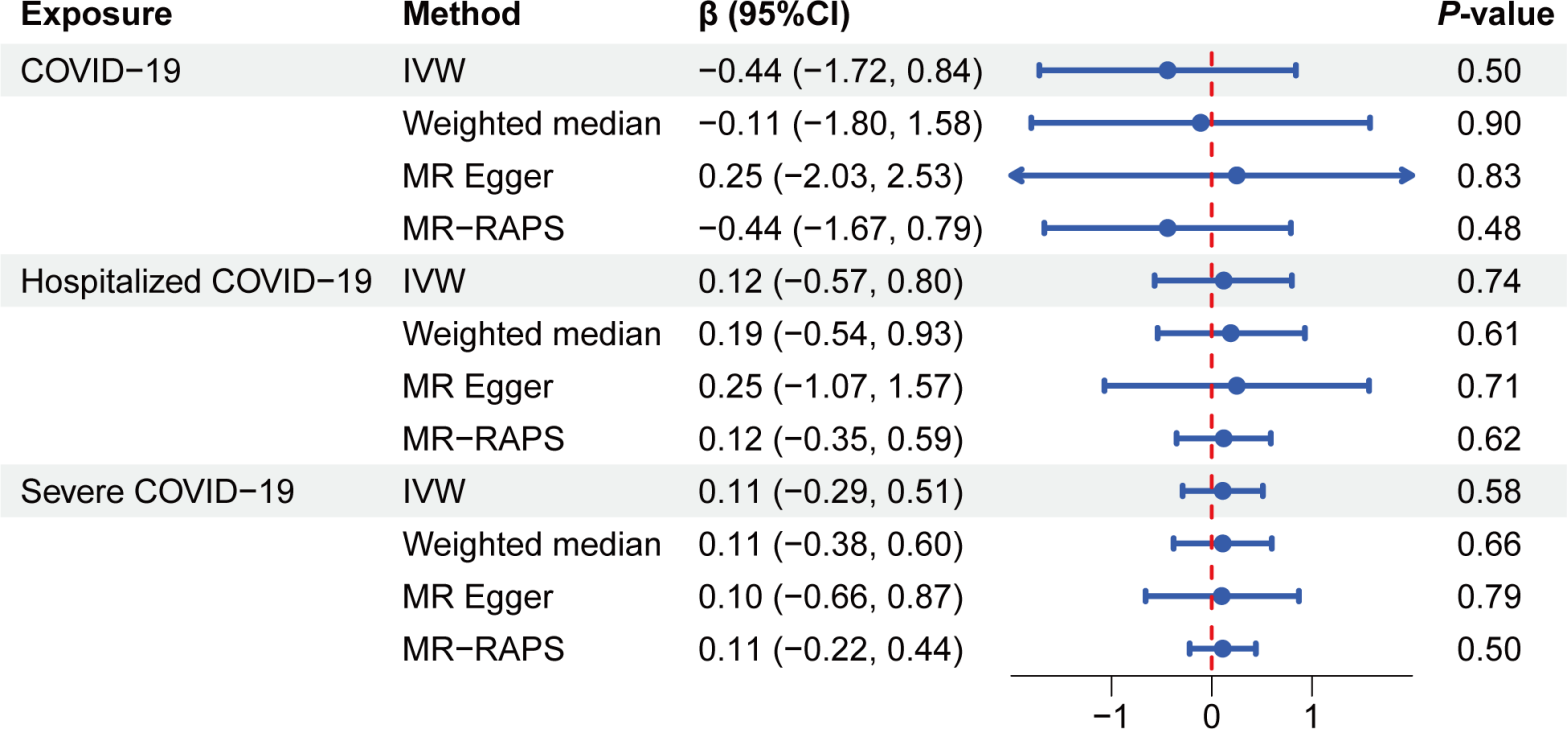
Forest plot of MR analysis results between three COVID-19 phenotypes and QT interval.

Following the robust validation provided by MRlap, which mitigated concerns about bias and sample overlap, we proceeded with univariate TSMR to assess the genetic causality of COVID-19 on QT interval. The IVW method revealed no significant associations between the three COVID-19 phenotypes (general infection, hospitalization, and severe cases) and QT interval changes [COVID-19: β_IVW_ (95% CI): −0.44 (−1.72, 0.84), *P*=0.50; hospitalization: β_IVW_: 0.12 (−0.57, 0.80), *P*=0.74; severe case: β_IVW_: 0.11 (−0.29, 0.51), *P*=0.58] (Figure 1 and Supplementary Figure S2A). These findings suggest that there is no significant rg between any COVID-19 phenotype and QT interval changes. Consistent causal conclusions were observed across supplementary analyses, including weighted median [COVID-19: β (95% CI): −0.11 (−1.80, 1.58), *P*=0.90; hospitalization: β (95% CI): 0.19 (−0.54, 0.93), *P*=0.61; severe case: β (95% CI): 0.11 (−0.38, 0.60), *P*=0.66] and MR-Egger [COVID-19: β (95% CI): 0.25 (−2.03, 2.53), *P*=0.83; hospitalization: β (95% CI): 0.25 (−1.07, 1.57), *P*=0.71; severe case: β (95% CI): 0.10 (−0.66, 0.87), *P*=0.79] methods, which further supported the null association across all COVID-19 phenotypes (Figure 1 and Supplementary Figure S2A). The MR-RAPS score further confirmed the absence of a causal association [COVID-19: β_MR-RAPS_ (95% CI): −0.44 (−1.67, 0.79), *P*=0.48; hospitalization: β_MR-RAPS_: 0.12 (−0.35, 0.59), *P*=0.62; severe case: β_MR-RAPS_: 0.11 (−0.22, 0.44), *P*=0.50] (Figure 1).

β represents effect size and lines indicate a 95% confidence interval (95% CI). IVW: inverse variance weighting; MR-RAPS: MRMendelian randomization-robust adjusted profile score.

**Figure 2:**
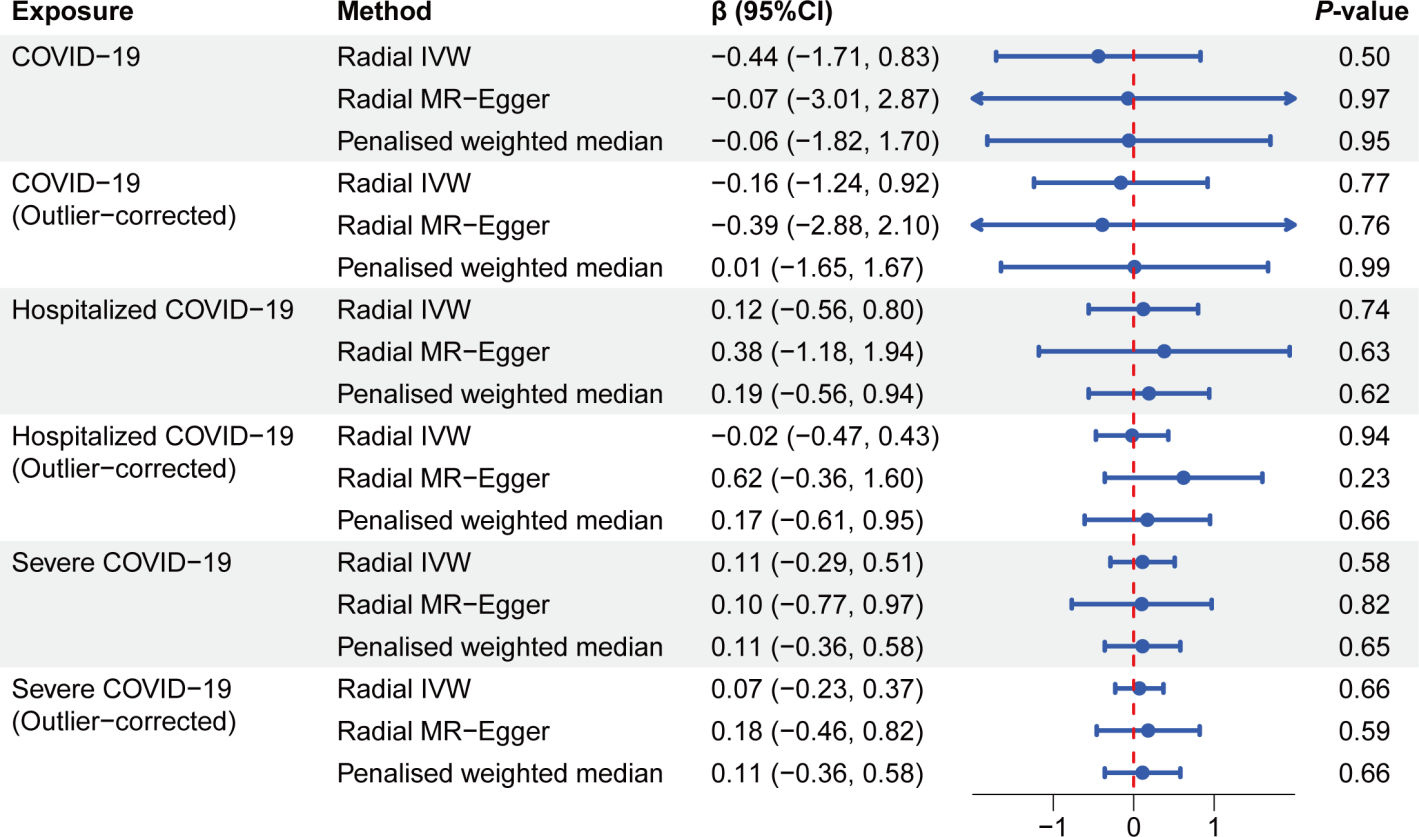
Forest plot of the results of radial MR and penalized weighted median MR analysis between the COVID-19 and QT interval.

**Figure 3:**
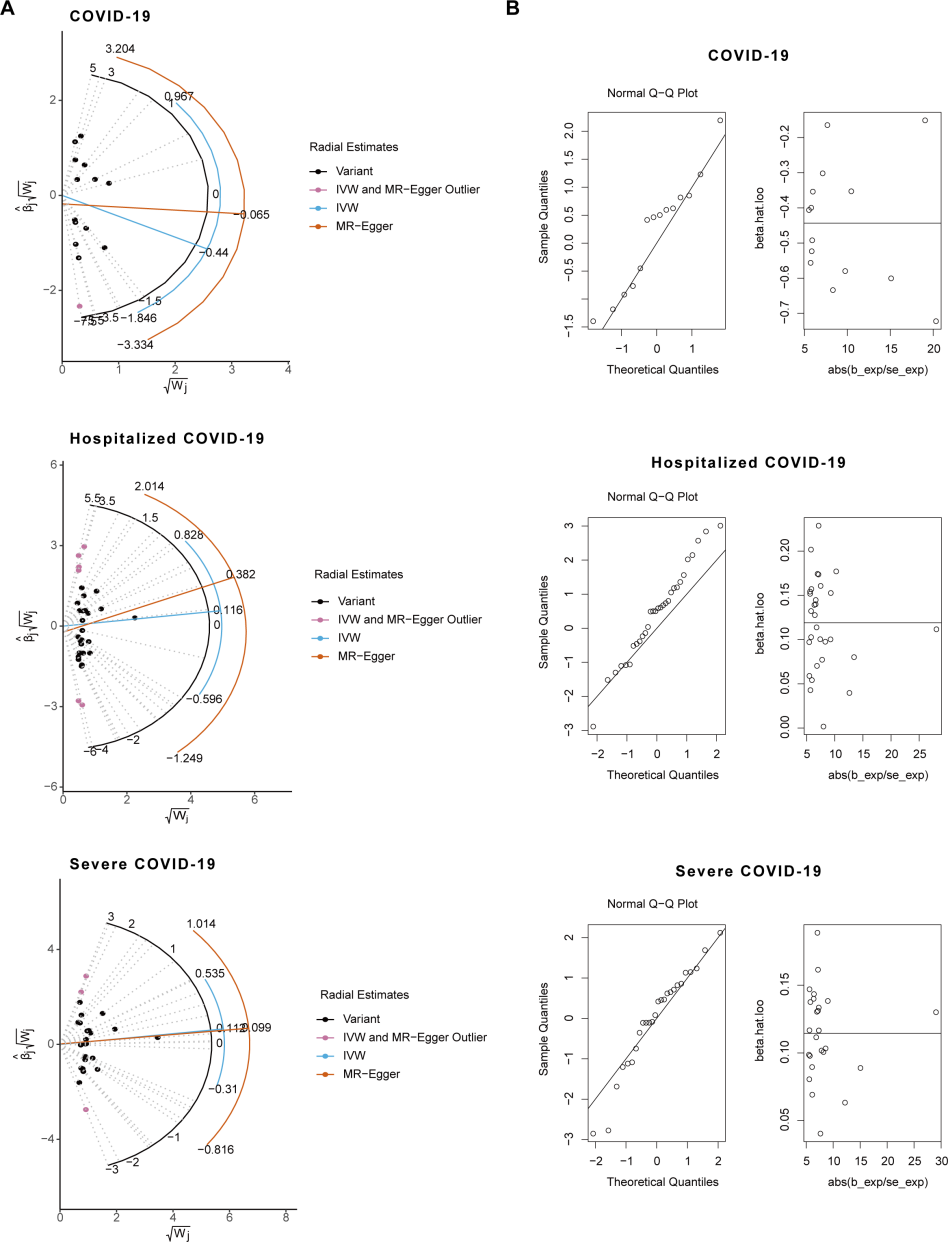
Radial MR plots and normality test plots for causality assessment between COVID-19 and QT interval.

The null causal estimates were similarly observed when using the penalized weighted median MR and in a radial MR framework. For radial IVW analysis, the estimates remained non-significant across the COVID-19 phenotypes [COVID-19: β_radial IVW_ (95% CI): −0.44 (−1.71, 0.83), *P*=0.50; hospitalization: β_radial IVW_: 0.12 (−0.56, 0.80), *P*=0.74; severe case: β_radial IVW_: 0.11 (−0.29, 0.51), *P*=0.58] (Figure 2). Radial MR plots identified outliers for each phenotype (one in COVID-19; six in hospitalization; and three in severe case; for QT*_Hof_* cohort, three in COVID-19; five in hospitalization; and three in severe case) (Table 2 and Figure 3A). Notably, corrective analyses conducted after removing these outliers did not alter the initial MR inferences, further supporting the robustness of our findings [COVID-19: β_radial IVW_ (95% CI): −0.16 (−1.24, 0.92), *P*=0.77; hospitalization: β_radial IVW_: −0.02 (−0.47, 0.43), *P*=0.94; severe case: β_radial IVW_: 0.07 (−0.23, 0.37), *P*=0.66] (Figure 2). These comprehensive analyses, encompassing various MR methods and sensitivity checks, consistently indicate that genetically determined COVID-19 susceptibility, hospitalization, and severity have no significant causal impact on QT interval changes.

The outliers are detected by radial IVW and radial MR-Egger methods, and the outlier-corrected analysis represents the analysis after the detected outliers are eliminated.

**Table 2:** Outliers in MR analysis identified by radial MR methods.

Exposure	Outcome	Outlier(s)
COVID-19	QT interval	rs11264339
Hospitalization		rs1634761, rs2102497, rs2897075, rs492602, rs63750417, rs78295726
Severe case		rs2897075, rs368565, rs62056905
COVID-19	QT interval (Hof)	rs11264339, rs184781326, rs679574
Hospitalization		rs63750417, rs492602, rs2326562, rs17412601, rs1634761
Severe case		rs62056905, rs368565, rs2236645

(**A**) Radial MR plots. The IVW and radial MR-Egger regression slopes calculated using first-order weights are superimposed, and radial curves highlight ratio estimates for each genetic variant and overall MR estimates. (**B**) Q plots for normality estimation.

### Direct causal effect for COVID-19 on QT interval adjusted by BMI, alcohol, and smoking

**Figure 4:**
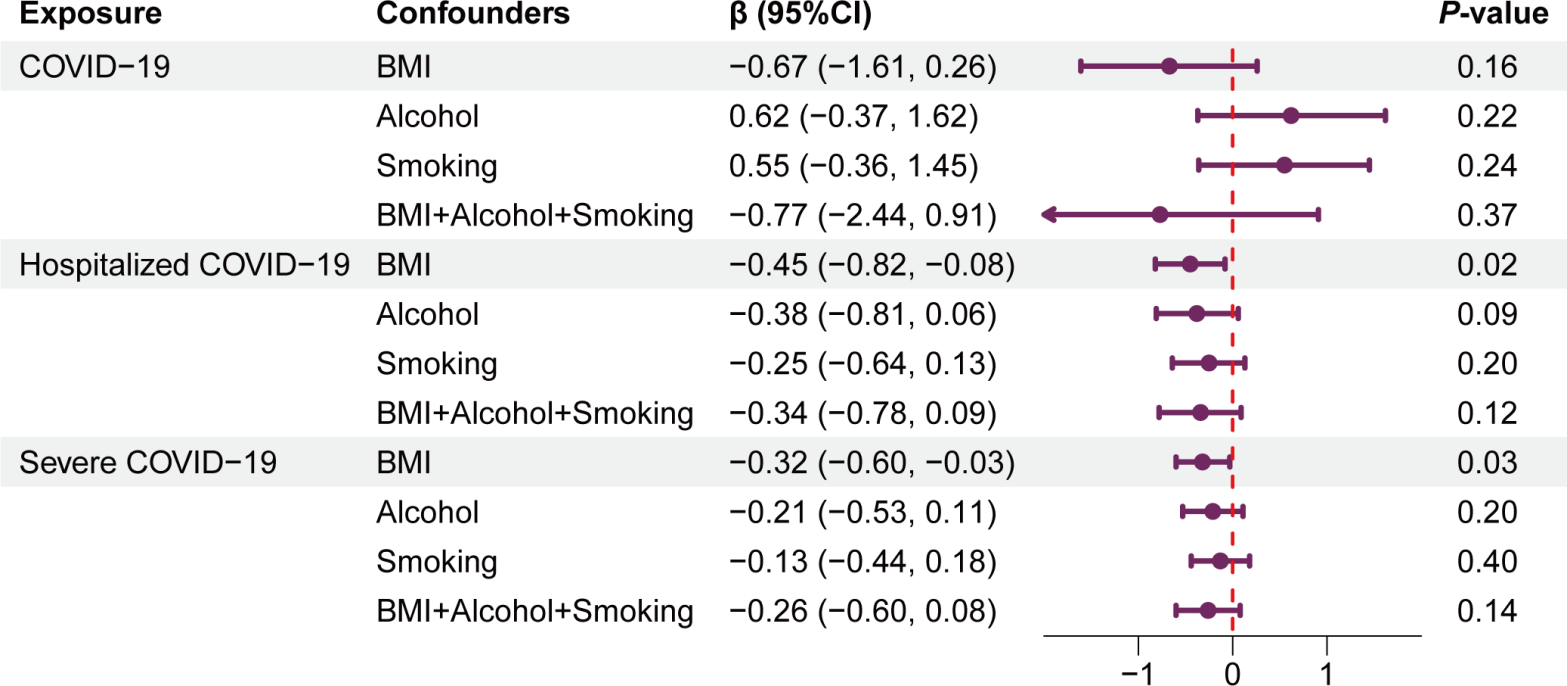
Forest plot of the results of the MVMR analysis for confounder correction.

To limit the potential pleiotropy, MVMR with BMI, alcohol, and smoking as covariates was also performed. Consistent with the findings of the UVMR, no significant causal effect was found between the multi-exposure-adjusted COVID-19 infection phenotype and prolonged QT interval in MVMR [β_BMI+Alcohol+Smoking_ (95% CI): −0.77 (−2.44, 0.91), *P*=0.37] (Figure 4). Similarly, after adjusting for smoking and alcohol consumption, the analysis of two other COVID-19 phenotypes also showed no significant MR results [hospitalization: β_Alcohol_ (95% CI): −0.38 (−0.81, 0.06), *P*=0.09, β_Smoking_: −0.25 (−0.64, 0.13), *P*=0.20; severe COVID-19: β_Alcohol_: −0.21 (−0.53, 0.11), *P*=0.20, β_Smoking_: −0.13 (−0.44, 0.18), *P*=0.40] (Figure 4).

BMI: body mass index, Alcohol: alcohol intake frequency, Smoking: smoking status.

Notably, in contrast with the null causal estimates observed in the UVMR analysis, the BMI-adjusted hospitalized and severe phenotypes exhibited a nominally significant shortening of the QT interval [hospitalization: β_BMI_ (95% CI): −0.45 (−0.82, 0.08), *P*=0.02; severe case: β_BMI_: −0.32 (−0.60,–0.03), *P*=0.03] (Figure 4). However, this significance did not reach the defined threshold for Bonferroni multiple corrections. Consequently, interpreting BMI as either existing within the pleiotropic pathway of genetic variation affecting the QT interval or as a mediator of causality from genetic variation through COVID-19 to the QT interval should be approached with caution.

### Validated causal estimates of COVID-19 on QT_(*Hof*)_ interval

To corroborate the consistency with the primary findings, additional QT interval GWAS data-QT(*_Hof_*) was employed for replication analysis. Similarly, MRlap analysis implied minimized sample overlap bias (Table 1). The causality estimates between genetically predicted exposures and outcomes in this analysis broadly recapitulated the primary study’s findings [COVID-19: β_IVW_ (95% CI): 1.68 (0.38, 2.99), *P*=0.01; hospitalization: β_IVW_: −0.01 (−0.61, 0.59), *P*=0.98; severe case: β_IVW_: −0.02 (−0.39, 0.34), *P*=0.91] (Supplementary Figures S2B and S3). For the COVID-19 infection phenotype, the IVW and MR-RAPS methods suggested a potential association with QT interval prolongation [COVID-19: β_IVW_ (95% CI): −1.68 (0.38, 2.99), *P*=0.01] (Supplementary Figure S3). However, other evaluation models did not support this conclusion, implying the possibility of false positives (Supplementary Figure S3). Moreover, the radial MR approach identified potential outliers in the analysis of the three cohorts, with 3, 5, and 3 outliers, respectively (Table 2 and Supplementary Figure S4A). Outlier-corrected radial MR did not reveal significant causal estimates in any of the COVID-19 cohorts (Supplementary Figure S3), underscoring the robustness of MR findings.

### Sensitivity analysis

**Figure 5:**
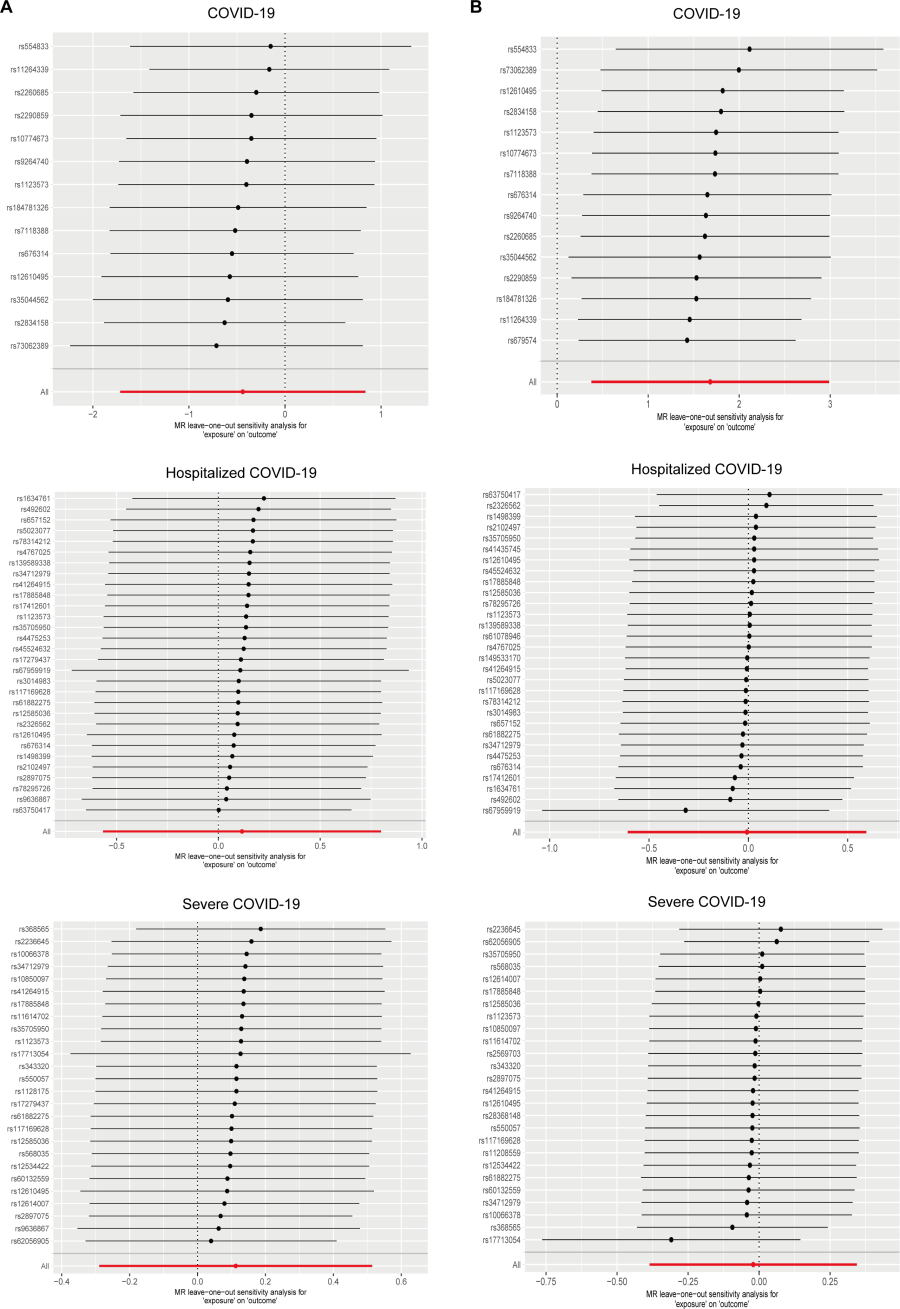
Mendelian randomization leave-one-out method analysis.

Heterogeneity assessment using Cochran’s Q test showed substantial heterogeneity for selected Is for almost all COVID-19 cohorts on QT intervals (*P*<0.05) (Table 3). This suggests variability in the genetic instruments that may be due to differences in the genetic architecture or environmental modifiers across cohorts. Despite this heterogeneity, the MR-Egger intercept did not show any significant departure from zero (*P*>0.05) (Table 3). This finding indicates a lack of pleiotropic bias in our analyses, implying that the associations observed are likely due to the specified exposures rather than unmeasured confounding. The lack of pleiotropic effects supports the validity of the IVs used in assessing the causal effects of COVID-19 on QT intervals. Furthermore, the quantile-quantile (Q–Q) plot for the MR-RAPS analysis confirmed adherence to the normality assumption required by the MR model (*P*>0.05), suggesting that the estimates converge to the true causal effect (Figure 3B and Supplementary Figure S4B). Leave-one-out analysis suggested that no single IV independently drove the overall MR effect, highlighting the consistency and reliability of our findings (Figure 5).

**Table 3:** MR sensitivity analysis results.

Exposure	Outcome	Heterogeneity (Cochrane's Q test)	Pleiotropy (MR Egger)		Normality (MR-RAPS)	
		*P*-value	Intercept	*P*-value	W statistics	*P*-value
COVID-19	QT interval	0.37	−0.05	0.48	0.94	0.40
Hospitalization		5.77E-04	−0.02	0.82	0.98	0.89
Severe case		0.05	0.00	0.98	0.95	0.18
COVID-19	QT interval (Hof)	0.01	0.06	0.37	0.94	0.37
Hospitalization		1.95E-05	−0.04	0.52	0.97	0.49
Severe case		0.01	−0.05	0.28	0.95	0.23

The red line represents the pooled IVWinverse variance weighted (IVW) estimator. (**A**) COVID-19 and QT interval. (**B**) COVID-19 and QT interval (*Hof*).

## Discussion

**Figure 6:**
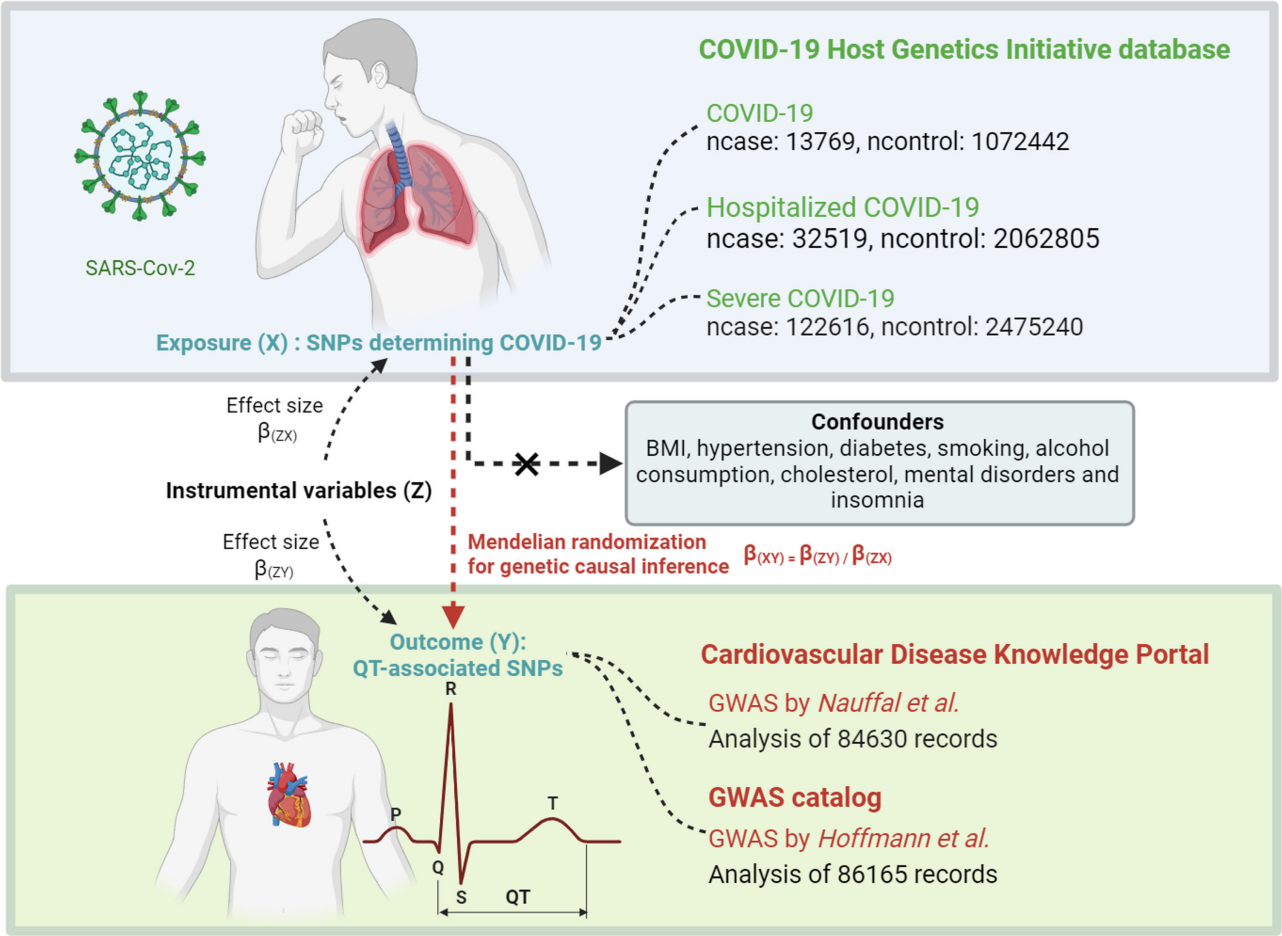
Mendelian randomization study investigating the causal relationship between COVID-19 (X) and QT interval prolongation (Y).

To address the constraints of confounding factors and reverse causality embedded in observational studies [30], we performed an MR analysis to investigate whether COVID-19 has a genetic causal impact on QT interval changes (Figure 6). In summary, our results did not favor that COVID-19 genetically accounts for longer or shorter QT intervals. These conclusions are derived from large GWAS sample datasets, multiple robust MR assessments, and confirmatory analyses. The discrepancy between our MR findings and previous observational studies may stem from several factors. First, observational studies are often challenged by confounding factors and reverse causality, which MR aims to minimize [30]. Moreover, QT interval measurements can be influenced by various non-genetic factors, including medication use, electrolyte imbalances, and individual physiological conditions [17,44].

Three COVID-19 phenotype GWAS datasets were used as exposures. Suitable genetic variants were selected as instrumental variables (Is, **Z**) for each dataset, ensuring their independence from potential confounders. The effects of these IVs on the exposure (β_ZX_) and outcome (QT interval, β_ZY_) were estimated. The causal effect of COVID-19 on QT interval prolongation (β_XY_) was inferred using the ratio of these two estimates, β_XY_ = β_ZY_ / β_ZX_.

While specific single-gene mutations can cause congenital long QT syndromes, the factors affecting acquired QT interval prolongation are multifaceted [45,46]. Recent GWAS studies have identified genetic loci associated with severe COVID-19, highlighting roles in inflammation, immune response, and clotting [28]. These findings provide novel insights into COVID-19-mediated multisystem complications. However, the complex biological pathways through which COVID-19 might induce arrhythmias, specifically QT interval prolongation, remain poorly understood. An MR analysis supports a causal link between serum calcium and prolonged QT interval [47], whereas it is unclear if COVID-19’s effect on the QT interval operates through changes in serum calcium concentration or other potential mediators affected by COVID-19. Our evidence suggests that COVID-19 does not directly influence QT interval changes, and, based on current understanding, much work remains to decipher which biomolecules or indicators play a mediating role in their correlation.

During a pandemic, multiple variables like myocarditis, electrolyte disturbances, hormonal changes, and autonomic nervous system dysfunction can influence QT interval [5,8,48,49]. Thus, SARS-CoV-2 infection is related to myocarditis and altered internal physiological factors [8]. Our MR analysis employs genetic variants of COVID-19 as the IVs may not fully account for these complex processes. Additionally, the multifaceted nature of COVID-19-induced inflammatory responses and multi-organ complications poses challenges for elucidating the pathogenic mechanisms of potential complications, including QT interval prolongation [1,6,12]. Furthermore, the association between COVID-19 and QT interval prolongation may involve undiscovered genetic or environmental factors.

COVID-19 is a complex and dynamic disease, and the timing of ECG measurements concerning the infection process may hold significant implications [13]. The impact of COVID-19 on QT intervals might vary with different stages of the disease, and our MR analysis did not adequately capture this temporal factor. To comprehensively understand the intricate relationship between COVID-19 and QT intervals, more datasets covering diverse population cohorts and different disease stages will provide deeper insights into the underlying genetic factors and potential mechanisms governing the association.

Furthermore, while our study found no genetic link between COVID-19 and QT interval prolongation, it does not exclude the potential for COVID-19 to elevate QT prolongation or arrhythmias risk. Disease GWASs are continually evolving to identify novel variants. For example, in the GWAS summary of QT intervals we included, despite meticulous consideration of polygenic risk-equivalent variants and single-gene putatively pathogenic rare variants, over 75% of individuals with markedly prolonged QT ( > 480 ms) did not exhibit these genetic variants [32]. This suggests that our understanding of the genetic factors influencing QT duration is limited and underscores the substantial influence of non-genetic factors on QT intervals.

This is the first study that examines the causal relationship between QT interval and COVID-19 through MR using the largest GWAS available for both exposure and outcome. Advanced and basic MR techniques were applied to estimate effects, and multiple sensitivity analyses confirmed the robustness of our results, providing largely unbiased causal estimates. Nonetheless, the study has its limitations. One notable constraint is the absence of data from non-European populations, thereby constraining the generalizability of the conclusions. Second, while MR techniques effectively reduce confounding bias, they are not entirely immune to it. The method assumes that genetic instruments are free from confounders and direct pleiotropic effects on the outcome, an assumption that may not always be fully accurate [30]. Third, MR analysis does not capture the temporal dynamics of COVID-19 and QT interval changes, as the impact of COVID-19 on the QT interval may vary across different disease stages, which genetic proxies may not fully reflect.

## Conclusion

Our MR assay revealed no causal relationship between COVID-19 and QT interval prolongation, indicating that observational study discrepancies may stem from residual confounding factors.

## Supplementary material

Online supplementary material 1

## Data Availability

All supporting data for this present study can be found within the article and its supplementary content.

## Abbreviations

BMI: Body mass index
CVDKP: Cardiovascular Disease Knowledge Portal
ECG: electrocardiogram
GWAS: genome-wide association study
IVW: inverse variance weighted
IVs: instrumental variables
LD: linkage disequilibrium clumping
MR: Mendelian randomization
MRCIEU: Medical Research Council Integrative Epidemiology Unit
MR-RAPS: Mendelian randomization-robust adjusted profile score
MVMR: multivariate Mendelian Randomization
SARS-CoV-2: severe acute respiratory syndrome coronavirus 2
SNPs: single nucleotide polymorphisms
TSMR: two-sample Mendelian randomization
