# Supplementary material for: Does COVID-19 impact the QT interval prolongation? Answers from genetic causal inference
Source: Biosci Rep. 2025 Jan 22;45(1):BSR20241281. doi: 10.1042/BSR20241281 (PMC12096949; doi:10.1042/BSR20241281)
Supplement: Online supplementary material 1 [file bsr-45-01-bsr-2024-1281-s001.docx]

# Using Mendelian randomization to unveil the truth: does COVID-19 impact QT interval prolongation?

**SUPPLEMENTARY MATERIAL**


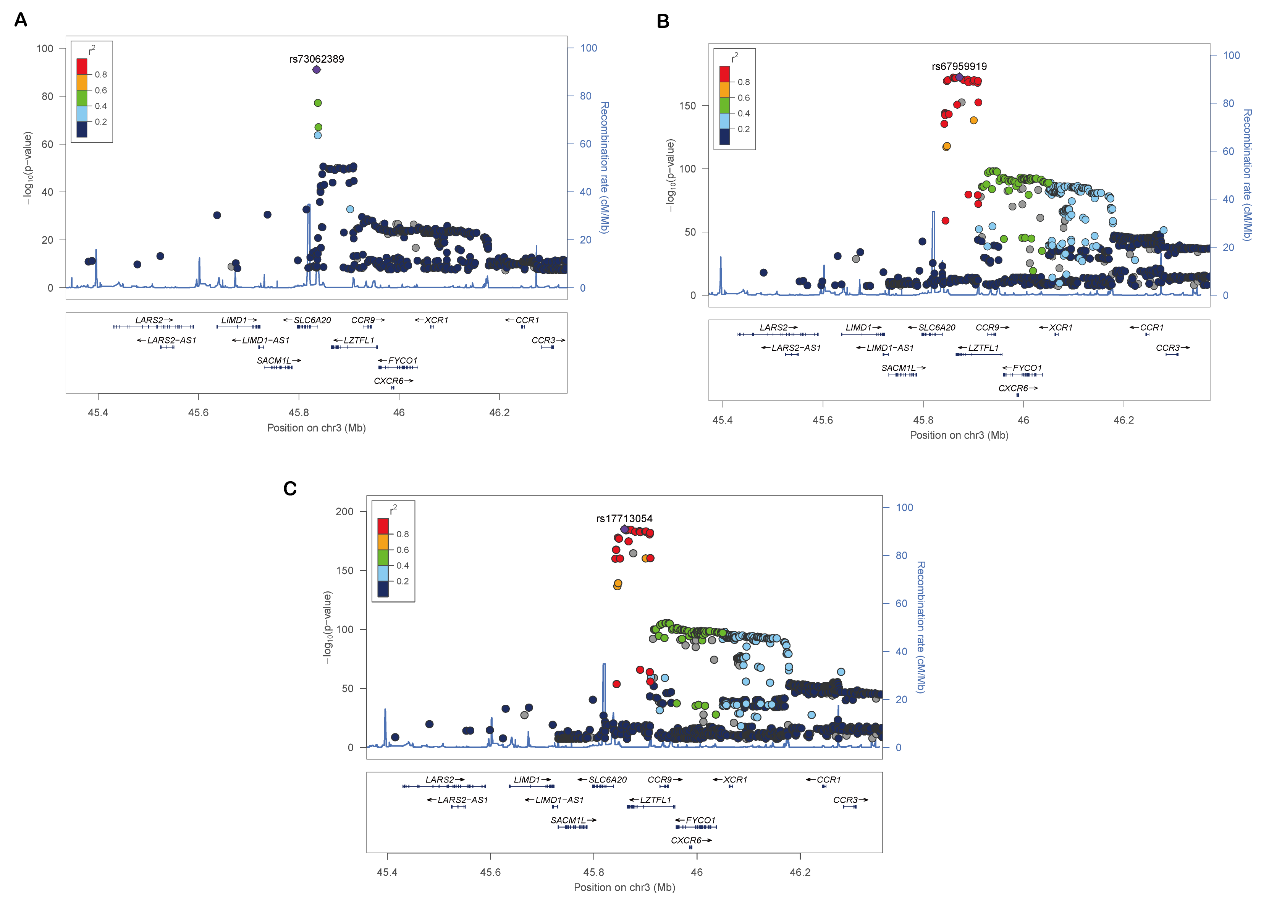


## Figure S1: Regional association plots on chromosome 3. The -log10(p-values) of single nucleotide polymorphisms (SNPs) (*P* < 5 × 10^-8^) in the three COVID-19 datasets are plotted based on their chromosomal location (lead SNP ± 500kb). The list of genes in the specified regions is presented below. (A) Regional association plot for lead SNPs rs73062389 in COVID-19. (B) Regional association plot for lead SNPs rs67959919 in hospitalized COVID-19. (C) Regional association plot for lead SNPs rs17713054 severe COVID-19.


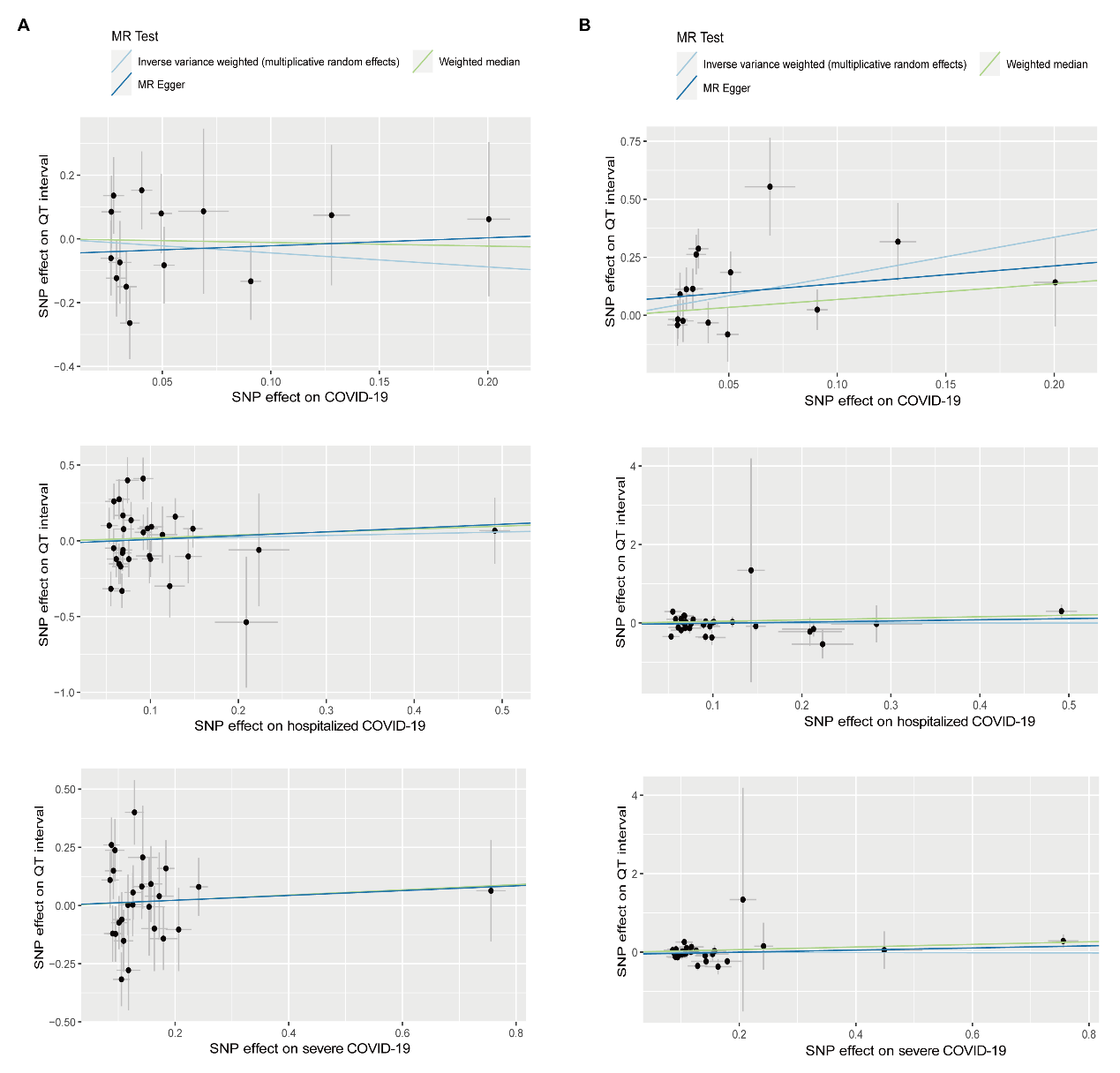


## Figure S2: Scatterplot for MR-estimated individual variant regression coefficients. (A) COVID-19 on QT interval. (B) COVID-19 on QT interval (*Hof*).


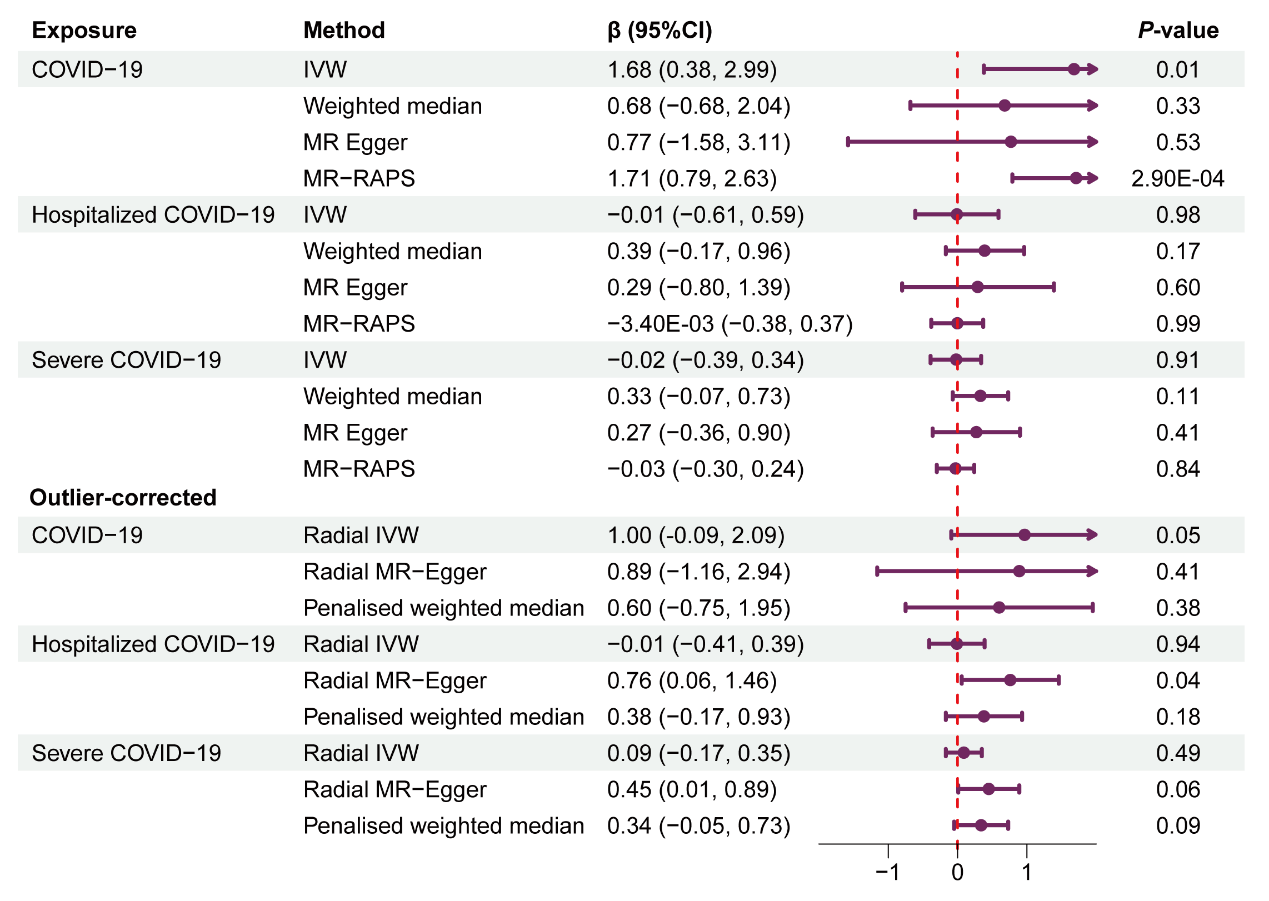


## Figure S3: Forest plot of MR analysis results between COVID-19 and QT interval (*Hof*) before and after outlier correction.


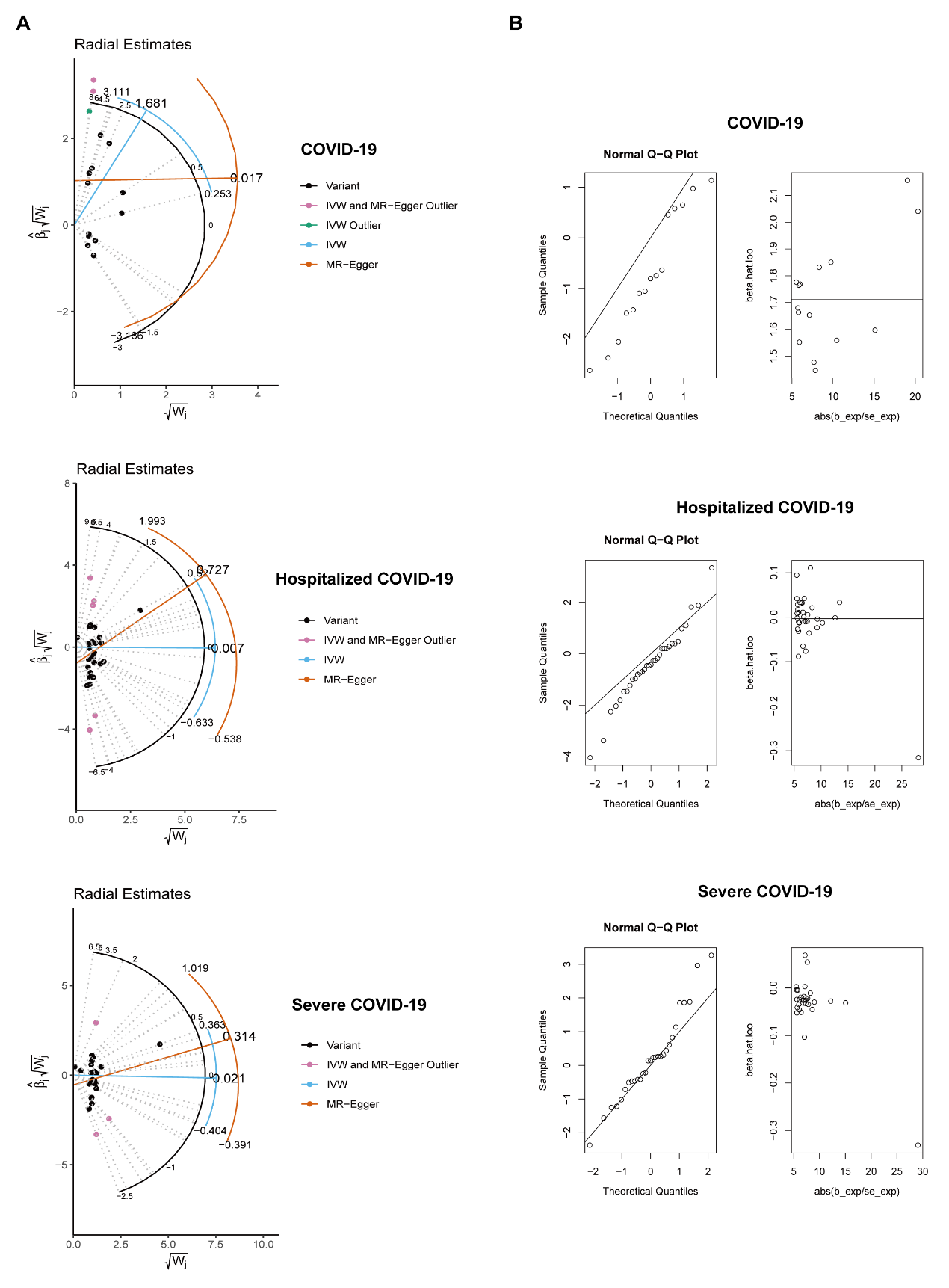


## Figure S4: Radial MR plots and normal distribution test plots between COVID-19 and QT interval (*Hof*). (A) Radial MR plots. The radial curves highlight the ratio estimates for each genetic variant and the overall MR estimates using the IVW calculated with first-order weights and the radial MR-Egger regression slopes superimposed. (B) Q-Q plot of normal estimates.
